# Prevalence of HPV infection and anal and cervical cytological abnormalities in transgender people at a referral service in Vitória, Espírito Santo state, Brazil, between 2018 and 2021

**DOI:** 10.1590/S2237-96222024v33e2024279.especial.en

**Published:** 2024-12-16

**Authors:** Franco Luís Salume Costa, Neide Aparecida Tosato Boldrini, Caroline Simões Caldeira, Carolina Loyola Prest Ferrugini, Lays Paula Bondi Volpini, Fenísia Gabrielle Carvalho Saldanha, Lucas Delboni Soares, Angelica Espinosa Miranda

**Affiliations:** 1 Universidade Federal do Espírito Santo, Programa de Pós-Graduação em Doenças Infecciosas, Vitória, ES, Brazil; 2 Universidade Federal do Espírito Santo, Departamento de Ginecologia e Obstetrícia, Vitória, ES, Brazil; 3 Universidade Federal do Espírito Santo, Vitória, ES, Brazil; 4 Universidade Federal do Espírito Santo, Programa de Pós-Graduação em Saúde Coletiva, Vitória, ES, Brazil

**Keywords:** Prevalencia, Personas transgénero, VPH, Chlamydia Trachomatis, Trichomonas Vaginalis, Prevalence, Transgender People, HPV, Chlamydia Trachomatis, Trichomonas Vaginalis

## Abstract

**Objectives:**

The aim of this study was to determine the prevalence of HPV and cytological alterations in the transgender population and contribute to the development of public policies.

**Methods:**

A descriptive study was conducted in a transgender outpatient clinic in Vitória, Espírito Santo state, between 2018 and 2021. Data were collected through interviews and information from medical records. Anogenital samples were collected for HPV, trichomoniasis, gonococcus and chlamydia testing, cytology.

**Results:**

Of the 110 participants, 60.9% identified as men and 34.5% as women. The overall prevalence of HPV was 58.3%, being higher in women (48.1%). Among men, cervical HPV was positive in 38%, and anal HPV in 25%, with cytological abnormalities found in 9.5%. Abnormal anal cytology was observed in 23.5% of women. Other sexually transmitted infections: chlamydia (4.1%), trichomoniasis (12.5%) and no cases of gonorrhea.

**Conclusion:**

HPV is a prevalent infection with risks for cytological abnormalities in the transgender population, and further studies on prevalence and impacts on sexual health are needed to support screening and prevention policies.

## Study contributions

**Main results:**

Transgender people are susceptible to sexually transmitted infections, with a high prevalence of HPV and, consequently, present cytological abnormalities in the anal and cervical regions, which pose a risk for the development of anogenital neoplasia.

**Implications for services:**

This is an invisible population, often absent from health services due to prejudice and stigma. Data on the health of this population contributes to the formulation of inclusive policies for the prevention, screening and diagnosis of STIs and anogenital neoplasia .

**Perspectives:**

Increased support are for further studies on STIs and anogenital neoplasia in the transgender population is needed, through investments in specialized outpatient clinics and research groups, so that public policies can be discussed and implemented.

## RESUMEN

**Objetivos:**

El objetivo de este estudio es determinar la prevalencia del VPH y cambios citológicos en la población transgénero, cooperar a la creación de políticas públicas.

**Métodos:**

Se realizó estudio descriptivo en un centro especializado en Vitoria-ES entre 2018-2021. Los datos se obtuvieron vía entrevistas y expedientes médicos. Se recogieron muestras anogenitales para investigación de VPH, tricomoniasis, gonorrea, clamidia, y citología.

**Resultados:**

De 110 participantes, 60,9% y 34,5% se identificaron como hombres y mujeres, respectivamente. Se encontró una prevalencia general de VPH de 58,3%, significativamente mayor en mujeres (48,1%). En hombres, el VPH cervical fue positivo en 38%, el VPH anal en un 25%, con cambios citológicos en 9,5%. Las mujeres con citología anal alterada representaron 23,5%. Otras infecciones de transmision sexual: clamidia (4,1%), tricomoniasis (12,5%), ningún caso de gonorrea.

**Conclusión:**

VPH es una infección prevalente con riesgos de alteraciones citológicas en esta población. Se necesitan más estudios sobre prevalencia e impactos en la salud sexual para fundamentar políticas de detección y prevención.

## INTRODUCTION

The concept of *transgenderism* is related to identity, while the term *gender* comes from a social construct. Thus, *gender identity* is an individual’s perception of themselves, which may or may not correspond to the sex assigned at birth.^1^ Gender identity is a determinant of health and poses significant challenges in accessing healthcare services.^2^

In this context, for the transgender population, health promotion requires a specific approach that goes beyond conventional care. Mitigating health disparities requires coordinated efforts involving healthcare professionals, educators, policymakers and society in general.^3^ Furthermore, limited access to sexual and reproductive health services is a concern, as inclusive approaches that respect gender identity diversity are necessary to ensure access to oral contraceptive pills, family planning, and care during gender transition.^2,4^ Globally, it is estimated that transgender people represent 0.5% to 1.3% of the population, and in Brazil, this estimate reaches 0.7% for transgender people and 1.2% for non-binary people.^5,6^

Studies addressing sexual and reproductive health, including the social determinants of illness and sexually transmitted infections (STIs), have not filled the knowledge gaps yet. These topics are essential as STIs are a global health problem, exacerbated in marginalized and stigmatized populations, such as the transgender population, where screening strategies are not recognized by clinical protocols or management guidelines.^2,7^ However, most data focus on HIV research in transgender women, with a lack of data regarding other STIs and their impact on the health of transgender people.^7,8^

Among STIs, HPV infection is the most prevalent among adults, with many cases being asymptomatic, in some cases presenting as warts or neoplasms of the anogenital region.^9^ These clinical manifestations of HPV are more common in groups of people associated with higher-risk behaviors, such as transgender people. ^10^ In Brazil, a study of the general population described a prevalence of 53.6% for HPV; of which, 35.2% tested positive for high-risk HPV for neoplasia, with 54.6% found in the female population, and 51.8% in the male population.^11^ No official data are available for the transgender population in the country. Information on trichomoniasis, chlamydia, and gonorrhea among transgender people is also scarce; though some studies report prevalence rates of up to 24.7% and 19.1% for chlamydia and gonorrhea, respectively.^8,12^ In order to contribute to the implementation of effective public policies for prevention and screening for the transgender population, the objective of this study was to determine the prevalence of STIs (anal and cervical HPV infection, trichomoniasis, gonorrhea and chlamydia) in the transgender population and the association between HPV and pre-neoplastic changes in anal and cervical cytology.

## METHODS

A cross-sectional study was conducted with transgender individuals receiving care at a specialized outpatient clinic for the transgender population in Vitória, Espírito Santo state, between August 2018 and May 2021. Those who agreed to take part in the study were included after signing the Free and Informed Consent Form. Data collection was performed through face-to-face interviews, containing sociodemographic, epidemiological, and general clinical data, as well as gender-specific information based on the individual’s self-identified gender. The interviewers were trained and were all part of the research team.

For data organization and analysis, transgender men and non-binary people were grouped together due to similarities in biological sex at birth. The demographic variables were as follow: age, categorized as “up to 24 years old”, “25 to 34 years old”, “35 to 44 years old” and “> 45 years old”; schooling, in years of study (4 to 9, 10 to 12 and more than 12); self-reported race/skin color (White, mixed-race, Black, Indigenous or Asian); and sexual orientation (heterosexual, homosexual, bisexual and other orientations).

Behavioral variables, such as alcohol use, engagement in sex worker, and experience of sexual assault, were dichotomized as “yes” or “no.” Sexual partners both lifetime and in the last year were categorized using the same modified methodological criteria of Boldrini.^13^ Regarding the coitarche, sexual intercourse was considered as any intimate act classified by the individual as sexual intercourse, with or without penetration, categorized as “yes” or “no” for any site. For anogenital site (anal or cervical), only penetration with a penis was considered, even though some people reported using penile prosthesis.

With regard to screening methods for neoplasia, it was taken into consideration conventional cervical cytology and HPV molecular test performed on transgender men, and conventional anal cytology and HPV molecular test performed on transgender women. The classification of cervical and anal cytological abnormalities followed the terminology proposed by the Brazilian nomenclature for cervical cytology reports, based on the Bethesda system, which applies the same cervical criteria to anal cytology.^30^ Thus, any cytological result other than “negative for intraepithelial lesion or cancer” or “unsatisfactory for evaluation” was considered abnormal cytology.^14^

Biological samples from the cervical and anal sites were collected for cytology and molecular biology analysis was performed through DNA extraction and identification of HPV, chlamydia, trichomoniasis and gonorrhea using polymerase chain reaction (PCR – RFLP) by trained medical professionals.^15^ Samples were only collected from individuals who had previous sexual intercourse and consented to the procedure. When abnormalities suggestive of malignancy were detected, colposcopy and anoscopy were performed by trained medical professionals and, if necessary, individuals with such abnormalities underwent biopsy using a Professor Medina biopsy punch, 3 mm in diameter, with the sample sent for anatomopathological analysis.

The information was categorized and stored anonymously using SPSS, version 20. For qualitative variables, descriptive analysis was performed, including frequency distribution, and mean and standard deviation (SD) were calculated for quantitative variables. The prevalence of abnormal cytology was determined by confirming the diagnosis, through cytopathological examination, and the corresponding 95% confidence interval (95%CI) was calculated. Associations with demographic variables were tested using the chi-square test, with Yates’ correction or Fisher’s exact test, as appropriate.

This study was approved by the Research Ethics Committee of the Hospital Universitário Cassiano Antonio Moraes, opinion number 3,442,602/2019. The information collected from the participants was used exclusively for research purposes, with confidentiality and data protection ensured at all times, including the signed consent forms and test results that could serve as a source of identification. The participants diagnosed with health conditions received counseling and were treated in accordance with the recommendations proposed by the Ministry of Health’s Clinical Protocol and Therapeutic Guidelines.^16^

## RESULTS

As a referral service offering various medical specialties, 572 users received care in at least one of these specialties by the end of the data collection period. Of these, only 110 agreed to participate in the study; the others either discontinued multidisciplinary follow-up in the multidisciplinary, declined to participate in the study or could not be contacted. Transgender men and non-binary people comprised the majority of the sample (n = 72; 65.5%), while transgender women accounted for 34.5% (n = 38).

The prevalence of HPV infection found in this study was 58.3%. Regarding sexual orientation, participants self-identified as heterosexual (72.7%), homosexual (4.5%) and bisexual (9.1%). Age ranged from 17 to 61 years, with a mean age of 27.7 years (standard deviation 9.143). Behavioral data by gender identity are reported in Table 1.

**Table 1:** Sociodemographic, clinical and behavioral data, by gender identity (n = 110)

Clinical behavioral data	Transgender men and non-binary people	Transgender women	p-value
**Age**			-
Up to 24 years old	41 (56.9)	13 (34.2)	
25-34 years old	26 (36.1)	10 (26.3)	
35-44 years old	5 (6.9)	9 (23.7)	
> 45 years	0	6 (15.8)	
**Schooling (years)**			0.029
4 to 9	8 (11.1)	12 (31.6)	
10 to 12	47 (65.3)	20 (52.6)	
> 12	17 (23.6)	6 (15.8)	
**Race/skin color**			**-**
Non-White	42 (58.3)	28 (73.7)	
White	30 (41.7)	10 (26.3)	
Alcohol use	36 (50.0)	8 (21.1)	0.003
**Sexual partnerships over lifetime**			0.040
Up to 5	27 (37.5)	13 (34.2)	
5 to 20	40 (55.6)	16 (42.1)	
More than 20	5 (6.9)	9 (23.7)	
**Sexual partnerships in the year**			0.303
Up to 5	67 (93.1)	33 (86.8)	
5 to 20	4 (5.6)	5 (13.2)	
More than 20	1 (1.4)	0 (0)	
**Denies condom use over lifetime**	40 (55.6)	12 (31.6)	0.017
**Never underwent screening for cervical/anal neoplasia**	48 (66.7)	38 (100)	< 0.001
**Sexual violence**	15 (20.8)	12 (31.6)	0.213
**Received money for sex**	5 (6.9)	10 (26.3)	0.005
**Intercourse with vaginal penetration**	41 (56.9)	0 (0)	< 0.001
**Intercourse with anal penetration**	14 (19.4)	34 (89.5)	< 0.001

**Figure 1:**
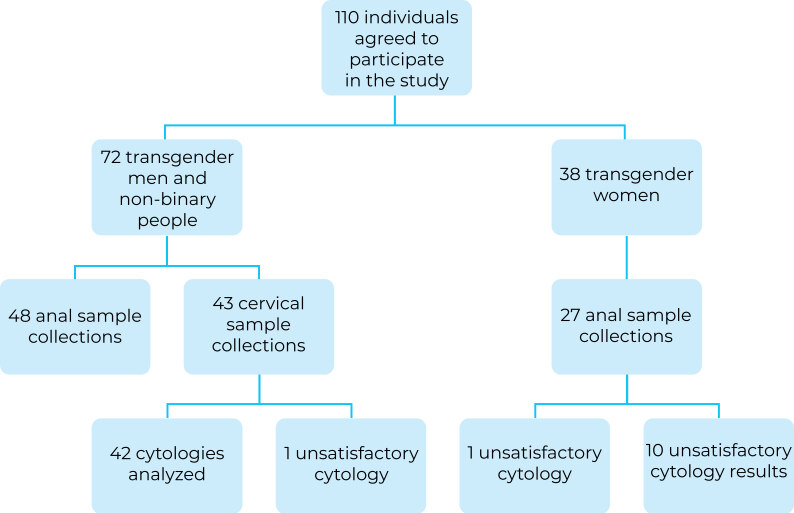
Samples from anogenital sites for HPV testing

Collection of anogenital samples for STI testing was subject to the participant’s refusal to undergo the procedure, as described in Figure 1.

Table 2 shows the results of HPV testing. The prevalence of HPV in transgender men, by anogenital site, in the cervical region, was 38%, and, in the anal region, 25%. Concurrent positive results for both anal and cervical sites were found in 11.6% of the total of 43 transgender men who had samples collected from both cervical and anal sites. Among the transgender women, 48.1% tested positive for HPV in the anal region. Regarding the type of HPV, samples from two users were tested, and types 33, 44, 52 and 62 were found, with types 33 and 52 classified as high-risk.

**Table 2:** Prevalence of positive HPV PCR results by collected samples, according to gender identity

HPV PCR in anal samples from transgender men and non-binary people (n = 48)	
HPV positive	11 (22.9%)
HPV negative	37 (77.1%)
**HPV PCR in cervical samples from transgender men and non-binary people (n = 43)**	
HPV positive	16 (37.2%)
HPV negative	27 (62.8%)
**HPV PCR in anal samples from transgender women (n = 27)**	
HPV positive	13 (48.1%)
HPV negative	14 (51.9%)

Table 3 highlights the prevalence of cytological abnormalities by anogenital site, with transgender women being the most affected with anal pre-neoplastic lesions in 23.5% of the samples. Transgender men showed cervical abnormalities in 9.5% of the samples. No anal cytological abnormalities were found in transgender men. All transgender women with abnormal anal cytology had a report with low-grade squamous intraepithelial lesion (LSIL). Other cytological abnormalities found in transgender men included: atypical squamous cells of undetermined significance (ASC-US), which could not rule out high-grade lesion (ASC-H), not excluding high-grade squamous intraepithelial lesion (HSIL). Unsatisfactory results for anal cytology were found in 6.4% of transgender women, and 13.6% of transgender men. In the total sample of participants, this result rate was 20%. Analyzing by number of collections, unsatisfactory rates were 29% among transgender women and 37.5% among transgender men.

**Table 3:** Prevalence of anogenital cytology results by collected samples, according to gender identity

Cervical cytology in transgender men and non-binary people (n = 42)	
Abnormal	4 (9.5%)
Normal	38 (90.5%)
**Anal cytology in transgender women (n = 17)**	
Abnormal	4 (23.5%)
Normal	13 (76.5%)

Participants with high-grade cytological lesions underwent site biopsy which confirmed the presence of high-grade lesions and were then referred for treatment through surgical excision of the transformation zone of the cervix – as per Brazilian guidelines for cervical cancer screening –,^17^ they are currently under follow-up. Table 4 shows clinical and cytopathological data, by cytological abnormalities, distributed by gender.

**Table 4:** Clinical and cytopathological data by cytological abnormalities, distributed according to gender

	Data	Macroscopic findings	HPV PCR results	Biopsy
**Cervical cytology (in transgender men)**				
ASC-US	30 years old, on hormone therapy	Extensive iodine-negative region	Negative	HSIL/CIN II and III
ASC-US	31 years old, on hormone therapy	Absence of abnormal findings	Negative	Not required
ASC-H	29 years old, on hormone therapy	Absence of abnormal findings	Negative	Not required
HSIL	18 years old, not on hormone therapy	Dense acetowhite area	Positive	HSIL/CIN III
**Anal cytology (in transgender women)**				
LSIL	32 years old, on hormone therapy	Absence of abnormal findings	Positive	Not required
LSIL	53 years old, on hormone therapy	Presence of anal warts	Positive	Not required
LSIL	19 years old, on hormone therapy	Presence of nonspecific papillomas	Positive	Not required
LSIL	39 years old, on hormone therapy	Presence of anal warts	Positive	Not required

The prevalence of other STIs at any anogenital site found in this study was 12.5% for trichomoniasis and 4.1% for chlamydia. No gonococcal infections were detected. When comparing chlamydia and trichomoniasis infection with behavioral data, non-use of condoms (p = 0.022), number of sexual partners in the past year above 20 (p = 0.028) and engaging in receptive anal sex (p = <0.001, were associated with diagnoses of chlamydia and trichomoniasis. Diagnoses of cervical HPV (p = <0.001) were related to chlamydia and trichomoniasis, indicating a higher risk for concomitant infection with these STIs in those sites. Both anal cytology and positivity for anal HPV were significant when stratified by gender identity (p-value 0.025 and 0.015, respectively). In the multivariate logistic regression analysis, HPV test positivity was considered the outcome variable, and no associations were observed in the final model with the following variables: age, schooling, condom use, number of lifetime sexual partners, engaging in anal and vaginal penetrative intercourse, undergoing screening cytology, and exchanging sex for money.

## DISCUSSION

This is the first study conducted in Espírito Santo that investigated the prevalence of HPV infection and abnormal anal and cervical cytology in transgender people receiving care at a specialized outpatient clinic. There is limited published data on HPV infection and cytological lesions in transgender people that are related to challenges in accessing health care for this population.^18^

The results showed a high prevalence of HPV infection (58.3%), similar to those of the study on HPV prevalence in Brazilian adults, presenting an overall prevalence of 53.6%, and to those of a study in transgender women, which found up to 77.9% of HPV positivity in the anal region.^11,19^ However, the findings of the present study differ from other studies conducted in the transgender population, which identified HPV prevalence of 19%, higher among transgender men (24.2%) compared to transgender women (11.8%).^20^ When analyzed by genital site, a higher presence of HPV in the anal site was observed in transgender women (48.1%) compared to transgender men, showing an increased risk of anal infections among women. Furthermore, HPV infection in the cervical site (37.2%) was consistent with previous studies that found cervical HPV up to 25%, but differs from other studies that identified positivity in 73% of people born with a cervix.^20,21^

There was no diagnosis of anal neoplasia, but abnormal oncotic cytology was found in 23.5% of transgender women – unlike what is described in other studies, which found rates of up to 91% –, aligning transgender women with groups at higher risk for anal neoplasia development.^22,23^ Nevertheless, the abnormal anal cytology found in this study is lower than that found in some cisgender populations (25.5%), especially among men who have sex with men (43.6%), but similar to heterosexual women (19.4%).^13^ The same occurs with the diagnosis of anal HPV, which is comparable to some cross-sectional studies in transgender women.^19^ In transgender men, cervical pre-neoplastic lesions did not influence the diagnoses of anal pre-neoplastic lesions, nor did the use of penile prostheses affect the diagnoses of cervical intraepithelial neoplasias or HPV in the cervical region.

Moreover, transgender women, who are traditionally associated, in the scientific literature, with high-risk behaviors and elevated rates of abnormal anal cytology, do not have specific protocols or guidelines for anal cancer screening.^10^ When asked about undergoing cytology tests for genital cancer screening, 78.2% of participants reported never having undergone one, while in transgender men this figure reached 66.7%, higher than in studies that identified 42.3% of absences in cervical cancer screening tests.^20^

In addition to the high frequency of HPV, it is worth noting that the prevalence of chlamydia infection is lower than that found in Brazilian studies, which identified 12% of this infection, and differs in the diagnoses of gonorrhea (6%), which was not identified in the cases evaluated in this study.^24^ Despite the prevalence of trichomoniasis reported in this study, recent studies on trichomoniasis positivity in the transgender population were found,^8,25^ making this information a pioneering finding in the study of STIs in the transgender population.

This study showed a higher proportion of transgender men in compared to transgender women, unlike what has been described in the medical literature.^26^ Nevertheless, STI diagnoses in transgender men were lower than those found in previous cross-sectional studies,^8^ though similar to other studies showing low diagnosis rates in this group,^12,26^ relating this fact to safe sexual behaviors among them, despite the high rate of non-use of condoms (55.6%). Regarding the impact of sexual behaviors on the prevalence of HPV, it could be seen that penile-penetrative intercourse in any site increases the likelihood of HPV infection; however, stratifying by site of penetration, the vaginal site shows a lower risk. Furthermore, the use of condoms does not protect against the transmission of the HPV, a fact well established by previous studies, which report the transmission of the virus even with condom use.^27^

Transgender people frequently face stigmatization and social discrimination, significantly impacting their well-being and health.^28^ Although nearly half of the sample reported accessing some health services prior to the research questionnaire, 46.2% reported some type of discrimination in care, mainly related to the disregard for their social name and gender identity. Another 44.2% stated that they did not feel adequately informed regarding gender transition in public health services. The lack of guidelines for welcoming and defining criteria for care and follow-up at the various healthcare levels for the transgender population remain a barrier, making it difficult to provide care for transgender people in health services and highlighting the need to break down barriers, deliver quality care and advance scientific understanding of this population.^29^

Difficulty in accessing healthcare and research centers hinders scientific research on the transgender population, impairing social inclusion actions and improvements in healthcare quality. It is crucial to create conditions for the implementation of effective and inclusive policies. Addressing the barriers faced by the transgender population in accessing healthcare services is essential to promote equity and improve health outcomes for this community.^2^ Implementing gender-sensitive and inclusive strategies is crucial to ensure that all individuals have access to quality healthcare, regardless of gender identity.^3^

This study had some limitations, such as the cross-sectional design which measures exposure and outcome simultaneously, thus preventing the establishment of a causal relationship; the sample size was also relatively small, which may have limited some comparisons and associations. In addition, the collection of anogenital samples was hindered by participants’ refusal, either due to lack of awareness of the importance of screening tests or because these tests could trigger dysphoric symptoms. Another limitation regarding anal site samples was the presence of unsatisfactory results due to the hypocellularity of the region, according to the updated Bethesda criteria, which hindered adequate histopathological analysis when looking for pre-neoplastic cellular abnormalities.^30^ Furthermore, the study was conducted during the COVID-19 pandemic, which suspended elective outpatient services, affecting the final sample size and contributing to missing data in research on anogenital samples and STIs. Despite these limitations, this study represents a significant advance in the health of the transgender population, given that it provides unprecedented exploratory data that can inform public policy formulations.

When transgender people are monitored in appropriate health services and receive care by trained health professionals, it is possible to advance policies for the prevention of anogenital tract cancer and STIs, thus increasing rates of appropriate diagnosis and early treatment. The high prevalence of genital HPV infection and abnormal anogenital cytology – even with primary and secondary prevention methods widely available in the Brazilian National Health System and broad knowledge in medical practice – is alarming and should draw the attention of public agencies and policies. Despite uncovering valuable data about this population, more robust studies are needed to better assess the prevalence of STIs and their association with clinical-behavioral data.
