# Supplementary material for: Prevalence of HPV infection and anal and cervical cytological abnormalities in transgender people at a referral service in Vitória, Espírito Santo state, Brazil, between 2018 and 2021
Source: Epidemiol Serv Saude. 2024 Dec 16;33(spe1):e2024279. doi: 10.1590/S2237-96222024v33e2024279.especial.en (PMC11654821; doi:10.1590/S2237-96222024v33e2024279.especial.en)
Supplement: Translation

# Translation: Prevalência da infecção pelo HPV e de alterações citológicas anais e cervicais em pessoas transgênero em um serviço de referência em Vitória no Espírito Santo entre 2018 e 2021

**DOI:** 10.1590/S2237-96222024v33e2024279.especial.pt

**Authors:** Franco Luís Salume Costa, Neide Aparecida Tosato Boldrini, Caroline Simões Caldeira, Carolina Loyola Prest Ferrugini, Lays Paula Bondi Volpini, Fenísia Gabrielle Carvalho Saldanha, Lucas Delboni Soares, Angelica Espinosa Miranda

**Affiliations:** 1 Universidade Federal do Espírito Santo, Programa de Pós-Graduação em Doenças Infecciosas, Vitória, ES, Brasil; 2 Universidade Federal do Espírito Santo, Departamento de Ginecologia e Obstetrícia, Vitória, ES, Brasil; 3 Universidade Federal do Espírito Santo, Vitória, ES, Brasil; 4 Universidade Federal do Espírito Santo, Programa de Pós-Graduação em Saúde Coletiva, Vitória, ES, Brasil

**Keywords:** Prevalência, Pessoas Transgênero, HPV, Chlamydia Trachomatis, Trichomonas Vaginalis

## Abstract

**Objetivos:**

O objetivo deste estudo é determinar a prevalência do HPV e de alterações citológicas na população transgênero e contribuir para a criação de políticas públicas.

**Métodos:**

Estudo descritivo conduzido em ambulatório transgênero em Vitória/ES, entre 2018 e 2021. O dados foram obtidos através de entrevista e informações de prontuário. Coletaram-se amostras anogenitais para a pesquisa de HPV, tricomoníase, gonococo e clamídia, e citologia.

**Resultados:**

Dos 110 participantes, identificaram-se como homens e mulheres, respectivamente, 60,9% e 34,5%. Encontrou-se prevalência geral de HPV de 58,3%, sendo maior em mulheres (48,1%). Em homens, HPV cervical foi positivo em 38%, e anal em 25%, com alterações citológicas em 9,5%. Mulheres com citologias anais alteradas foram 23,5%. Outras infecções sexualmente transmissíveis: clamídia (4,1%), tricomoníase (12,5%) e nenhum caso de gonorreia.

**Conclusão:**

O HPV é uma infecção prevalente e com riscos para alterações citológicas na população transgênero, sendo necessários mais estudos de prevalência e de impactos na saúde sexual, para basear políticas de rastreamento e prevenção.

## Contribuições do estudo

**Principais resultados:**

Pessoas transgênero são suscetíveis a infecções sexualmente transmissíveis, com alta prevalência de HPV e, por isso, apresentam alterações citológicas em sítio anal e cervical que trazem risco para o desenvolvimento de neoplasia anogenital.

**Implicações para os serviços:**

Trata-se de uma população invisibilizada e ausente dos serviços de saúde, por conta de preconceitos e estigmas. Dados sobre a saúde dessa população contribuem para a formulação de políticas inclusivas de prevenção, rastreio e diagnóstico de IST e neoplasia anogenital.

**Perspectivas:**

Esperam-se incentivos para mais estudos acerca de IST e neoplasia anogenital em população transgênero, por meio de investimentos em ambulatórios especializados e em grupos de pesquisa, para que possam ser debatidas e aplicadas políticas públicas.

## INTRODUÇÃO

O conceito de *transgeneridade* se refere a identidade, enquanto o termo *gênero* vem de uma construção social. Assim, *identidade de gênero* é uma percepção do indivíduo sobre si, que pode ser igual ou diferente do sexo designado ao nascimento.^1^ A identidade de gênero é um determinante de saúde e impõe desafios significativos no acesso aos serviços de saúde.^2^

Nesse sentido, para a população transgênero, a promoção da saúde exige uma abordagem específica que ultrapasse a assistência convencional. A mitigação das disparidades na saúde requer esforços coordenados, envolvendo profissionais de saúde, educadores, formuladores de políticas e a sociedade em geral.^3^ Além disso, a limitação do acesso pleno aos serviços de saúde sexual e reprodutiva é uma preocupação, pois abordagens inclusivas, que respeitem a diversidade de identidades de gênero, são necessárias para garantir o acesso a contraceptivos, planejamento familiar e cuidados durante a transição de gênero.^2,4^ No mundo, estima-se de 0,5% a 1,3% de população transgênero, e no Brasil, essa estimativa chega a 0,7% de pessoas transgênero e 1,2% de pessoas não binárias.^5,6^

Estudos que abordem a questão da saúde sexual e reprodutiva, incluindo os determinantes sociais do processo de adoecimento e infecções sexualmente transmissíveis (IST), ainda não preencheram as lacunas de conhecimento. Esses temas são importantes, pois as IST são um problema de saúde global, agravado para populações marginalizadas e estigmatizadas, como a população transgênero, em que as estratégias de rastreamento não são reconhecidas por protocolos ou guias de manejo clínico.^2,7^ No entanto, a maioria dos dados focam pesquisas de HIV em mulheres transgênero, havendo deficiência de dados em relação às outras IST e seus impactos na saúde de pessoas transgênero.^7,8^

Entre as IST, a infecção pelo HPV é a mais prevalente entre os adultos, sendo grande parte assintomática, em alguns casos podendo se apresentar como verrugas ou neoplasias de sítio anogenital.^9^ Essas manifestações clínicas do HPV são mais frequentes em grupos de pessoas mais associados a comportamentos de risco, como pessoas transgênero.^10^ No Brasil, um estudo na população geral descreveu uma prevalência de 53,6% de HPV; destes, 35,2% apresentaram positividade para HPV de alto risco para neoplasia, 54,6% encontrados na população feminina, e 51,8%, na população masculina.^11^ Não existem informações oficiais de população transgênero no país. Informações sobre tricomoníase, clamídia e gonorreia, entre as pessoas transgênero, também não são frequentes; alguns estudos descrevem taxas de prevalência de até 24,7% e 19,1% de clamídia e gonorreia, respectivamente.^8,12^ A fim de contribuir na implementação de políticas públicas de prevenção e rastreamento efetivas para a população transgênero, o objetivo deste estudo foi determinar a prevalência de IST (infeção pelo HPV anal e cervical, tricomoníase, gonorreia e clamídia) na população transgênero e a associação de HPV com alterações pré-neoplásicas em citologias de sítio anal e cervical.

## MÉTODOS

Estudo de corte transversal realizado com pessoas transgênero atendidas em um ambulatório especializado e de referência para a população transgênero em Vitória, no estado do Espírito Santo, entre agosto de 2018 e maio de 2021, que aceitaram participar da pesquisa e foram incluídas após assinatura do Termo de Consentimento Livre e Esclarecido. A coleta de dados foi realizada por meio de entrevista face a face, contendo dados sociodemográficos, epidemiológicos e clínicos em geral, e específicos para o gênero com o qual a pessoa se identificava. Os entrevistadores foram treinados e todos faziam parte da equipe de pesquisa.

Para a organização e análise de dados, por possuírem semelhanças quanto ao sexo biológico ao nascimento, homens transgênero e pessoas não binárias foram reunidos no mesmo grupo. As variáveis demográficas foram: idade, categorizada em “até 24 anos”, “25 a 34 anos”, “35 a 44 anos” e “> 45anos”; escolaridade, em anos de estudo (4 a 9, 10 a 12 e mais de 12); raça/cor da pele autodeclarada (branco, pardo, negro, indígena ou amarelo); e orientação sexual (heterossexual, homossexual, bissexual e outras orientações).

Variáveis comportamentais, como uso de álcool, se atuou como trabalhador(a) do sexo e se vivenciou agressão sexual, foram dicotomizadas em “sim” e “não”. Parcerias sexuais na vida e no último ano foram categorizadas seguindo-se os mesmos critérios metodológicos modificados de Boldrini.^13^ Para a pesquisa de coitarca, considerou-se relação sexual como qualquer ato íntimo classificado pela pessoa como relação sexual, com ou sem penetração, sendo consideradas em qualquer sítio as categorias “sim” ou “não”. Por sítio anogenital (anal ou cervical), considerou-se apenas penetração com pênis, mesmo algumas pessoas relatando uso de próteses penianas. 

Na pesquisa de métodos para rastreamento de neoplasia, considerou-se a realização de citologia oncótica tradicional de cérvice uterina e teste molecular de HPV em homens transgênero, e citologia oncótica tradicional de canal anal e teste molecular de HPV em mulheres transgênero. A classificação das alterações citológicas cervicais e anais seguiu a terminologia proposta pela nomenclatura brasileira para laudos cervicais, baseada no sistema Bethesda, que usa os mesmos critérios cervicais para citologias anais.^30^ Assim, são consideradas alterações em citologias a presença de qualquer resultado que não fosse “negativo para lesão intraepitelial ou câncer” ou “insatisfatório para avaliação”.^14^

Foi realizada coleta de amostras biológicas de sítio cervical de colo uterino e anal, para citologia, e análise de biologia molecular via extração de DNA e identificação de HPV, clamídia, tricomoníase e gonorreia através de reação em cadeia da polimerase (PCR – RFLP), por profissionais médicos treinados.^15^ As coletas somente foram realizadas em pessoas com relação sexual anterior e que consentiram em realizar o procedimento. Quando detectadas alterações sugestivas de malignidade, colposcopia e anuscopia foram realizadas por profissionais médicos treinados e, em caso de necessidade, as pessoas com tais alterações foram submetidas à biópsia com uma pinça do tipo Professor Medina, de 3 mm de sacabocado, e a amostra foi encaminhada para análise anatomopatológica. 

As informações foram categorizadas e armazenadas anonimamente, utilizando-se o SPSS, versão 20. Para variáveis qualitativas, realizou-se análise descritiva, incluindo distribuição de frequência, assim como cálculo de média e desvio-padrão (DP) para variáveis quantitativas. A prevalência de citologias alteradas foi determinada pela confirmação de diagnóstico, por meio do exame citopatológico, sendo calculado o correspondente intervalo de confiança de 95% (IC_95_%). As associações com as variáveis demográficas foram testadas por meio de teste qui-quadrado, com correção de Yates ou teste de Fisher, quando apropriado. 

O Comitê de Ética em Pesquisa do Hospital Universitário Cassiano Antonio Moraes aprovou este estudo, com o número do parecer 3.442.602/2019. As informações coletadas dos participantes foram utilizadas apenas para fins de pesquisa, com garantia de confidencialidade e proteção dos dados durante todo o tempo, incluindo-se os termos de consentimento assinados e os resultados de exames que possam servir como fonte de identificação. Os usuários diagnosticados receberam aconselhamento e foram tratados de acordo com as recomendações propostas pelo Protocolo Clínico e Diretrizes Terapêuticas do Ministério da Saúde.^16^

## RESULTADOS

Por se tratar de um serviço de referência com disponibilidade de diversas especialidades, 572 usuários foram atendidos em qualquer uma dessas especialidades até o término da recolha de dados. Desses, apenas 110 aceitaram participar do estudo; os demais perderam seguimento no acompanhamento multidisciplinar, não aceitaram participar do estudo ou deles não se conseguiu contato. Homens transgênero e identidade não binária foram a maioria da amostra (n = 72; 65,5%), enquanto as mulheres transgênero representaram 34,5% (n = 38). 

A prevalência de infecção pelo HPV encontrada no estudo foi de 58,3%. Em relação à orientação sexual, os participantes se autodeclararam heterossexuais (72,7%), homossexuais (4,5%) e bissexuais (9,1%). A idade variou entre 17 e 61 anos, sendo a média de 27,7 anos (desvio-padrão 9,143). Dados comportamentais por identidade de gênero estão relatados na Tabela 1.

**Tabela 1:** Dados sociodemográficos, clínicos e comportamentais, por identidade de gênero (n = 110)

Dados clínicos e comportamentais	Homens transgênero e não binários (n = 72) N (%)	Mulheres transgênero (n = 38) N (%)	p-valor
**Idade**			**-**
Até 24 anos	41 (56,9)	13 (34,2)	
25-34 anos	26 (36,1)	10 (26,3)	
35-44 anos	5 (6,9)	9 (23,7)	
> 45 anos	0	6 (15,8)	
**Escolaridade (anos)**			0,029
4 a 9	8 (11,1)	12 (31,6)	
10 a 12	47 (65,3)	20 (52,6)	
> 12	17 (23,6)	6 (15,8)	
**Raça/cor da pele**			-
Não branco	42 (58,3)	28 (73,7)	
Branco	30 (41,7)	10 (26,3)	
Uso de álcool	36 (50,0)	8 (21,1)	0,003
**Parcerias sexuais na vida**			0,040
Até 5	27 (37,5)	13 (34,2)	
5 a 20	40 (55,6)	16 (42,1)	
Acima de 20	5 (6,9)	9 (23,7)	
**Parcerias sexuais no ano**			0,303
Até 5	67 (93,1)	33 (86,8)	
5 a 20	4 (5,6)	5 (13,2)	
Acima de 20	1 (1,4)	0 (0)	
**Nega uso de preservativo na vida**	40 (55,6)	12 (31,6)	0,017
**Nunca realizou prevenção para neoplasia cervical/anal**	48 (66,7)	38 (100)	< 0,001
**Violência sexual**	15 (20,8)	12 (31,6)	0,213
**Recebeu dinheiro para ter relações sexuais**	5 (6,9)	10 (26,3)	0,005
**Relação com penetração vaginal**	41 (56,9)	0 (0)	< 0,001
**Relação com penetração anal**	14 (19,4)	34 (89,5)	< 0,001

As coletas de sítios anogenitais para pesquisa de IST foram condicionadas à recusa do participante quanto à realização do procedimento, conforme descrito na Figura 1. 

**Figura 1:** Amostras de sítios anogenitais para pesquisa de HPV

A Tabela 2 descreve os resultados dos testes para HPV. A prevalência de HPV em homens transgênero, por sítio anogenital, em região cervical, foi de 38%, e, em região anal, de 25%**.** Foram positivos em sítio anal e cervical concomitantemente 11,6%, no total de 43 homens transgênero com coletas em sítios cervical e anal. Entre as mulheres transgênero, 48,1% foram positivas para HPV em região anal. Em relação ao tipo do HPV, foram testadas amostras de dois usuários, e encontrados os tipos 33, 44, 52 e 62, sendo o 33 e o 52 considerados como de alto risco. 

**Tabela 2:** Prevalência de resultados positivos de PCR para HPV por amostras coletadas, segundo identidade de gênero

**PCR para HPV em amostras anais em homens transgênero e identidades não binárias (n = 48)**	
HPV positivo	11 (22,9%)
HPV negativo	37 (77,1%)
**PCR para HPV em amostras cervicais em homens transgênero e identidades não binárias (n = 43)**	
HPV positivo	16 (37,2%)
HPV negativo	27 (62,8%)
**PCR para HPV em amostras anais em mulheres transgênero (n = 27)**	
HPV positivo	13 (48,1%)
HPV negativo	14 (51,9%)

A Tabela 3 evidencia a prevalência de alterações citológicas por sítio anogenital, sendo que mulheres transgênero foram as mais afetadas com lesão pré-neoplásica anal em 23,5% das amostras. Homens transgênero apresentaram alteração no colo do útero em 9,5% das amostras. Não foram encontradas alterações citológicas anais em homens transgênero. Todas as mulheres transgênero com citologia anal alterada apresentaram laudo com lesão intraepitelial de baixo grau (LSIL). Outras alterações citológicas encontradas em homens transgênero foram: células escamosas atípicas de significado indeterminado (ASC-US), células escamosas atípicas de significado indeterminado, não podendo afastar lesão de alto grau (ASC-H), e neoplasia intraepitelial de alto grau (HSIL). Os resultados insatisfatórios para citologia anal foram em mulheres transgênero de 6,4%, e em homens transgênero, de 13,6%. Na amostra total de participantes, esse valor foi de 20%. Quando se analisa por número de coletas, temos 29% de insatisfatório entre as mulheres, e para homens transgêneros, de 37,5%.

**Tabela 3:** Prevalência de citologias anogenitais por amostras coletadas, segundo identidade de gênero

**Citologia cervical em homens transgênero e identidades não binárias (n = 42)**	
Alterada	4 (9,5%)
Não alterada	38 (90,5%)
**Citologia anal em mulheres transgênero (n = 17)**	
Alterada	4 (23,5%)
Não alterada	13 (76,5%)

Participantes com lesão citológica de alto grau foram submetidos à biópsia de sítio com confirmação de lesão de alto grau, e foram, logo em seguida, encaminhados para tratamento por exérese de zona de transformação do colo do útero – conforme diretrizes brasileiras para o rastreamento do câncer do colo de útero–,^17^ e seguem em acompanhamento. A Tabela 4 descreve dados clínicos e citopatológicos, por alterações citológicas, distribuídas por gênero. 

**Tabela 4:** Dados clínicos e citopatológicos por alterações citológicas, distribuídos segundo gênero

	Dados	Achado macroscópico	PCR para HPV	Biópsia
**Citologia cervical (em homens transgênero)**				
ASC-US	30 anos, em uso de hormônio	Área iodo negativa extensa	Negativo	HSIL/NICII e III
ASC-US	31 anos, em uso de hormônio	Ausência de achados anormais	Negativo	Não necessário
ASC-H	29 anos, em uso de hormônio	Ausência de achados anormais	Negativo	Não necessário
HSIL	18 anos, sem uso de hormônio	Área acetobranca densa	Positivo	HSIL/NICIII
**Citologia anal (em mulheres transgênero)**				
LSIL	32 anos, em uso de hormônio	Ausência de achados anormais	Positivo	Não necessário
LSIL	53 anos, em uso de hormônio	Presença de condilomas anais	Positivo	Não necessário
LSIL	19 anos, em uso de hormônio	Presença de papilomas de caráter inespecíficos	Positivo	Não necessário
LSIL	39 anos, em uso de hormônio	Presença de condilomas anais	Positivo	Não necessário

A prevalência de outras IST em qualquer sítio anogenital encontrada no estudo foi de 12,5% de tricomoníase e de 4,1% de clamídia. A infecção pelo gonococo não foi encontrada. Na comparação da infecção pela clamídia e tricomoníase com dados comportamentais, o não uso de preservativo (p = 0,022), o número de parceiros(as) sexuais no ano acima de 20 (p = 0,028) e a prática de sexo anal receptivo (p = < 0,001) foram associados aos diagnósticos de clamídia e tricomoníase. Diagnósticos de HPV cervical (p = < 0,001) apresentaram relação com clamídia e tricomoníase, indicando que existe maior risco para infecção concomitante entre essas IST nesses sítios. Tanto a citologia anal quanto a positividade para HPV anal apresentaram significância na estratificação por identidade de gênero (p-valor 0,025 e 0,015, respectivamente). Na análise multivariada de regressão logística, a positividade para o teste de HPV foi considerada como variável de desfecho, e não se observou associação no modelo final com as seguintes variáveis: idade, escolaridade, uso de preservativo, número de parcerias sexuais na vida, realização de sexo com penetração anal e vaginal, realização de alguma citologia de rastreamento e troca de sexo por dinheiro.

## DISCUSSÃO

Este é o primeiro estudo conduzido no Espírito Santo que pesquisou a prevalência da infecção pelo HPV e de citologias anais e cervicais alteradas em pessoas transgênero atendidas em ambulatório especializado. Há poucos dados publicados sobre infecção pelo HPV e lesões citológicas em pessoas transgênero que se relacionam com dificuldades no acesso à saúde dessa população.^18^

Os resultados mostraram alta prevalência de infecção pelo HPV (58,3%), resultados similares ao do estudo de prevalência de HPV em adultos brasileiros, que obteve prevalência geral de 53,6%, e ao de estudo em mulheres transgênero que encontrou até 77,9% de positividade para HPV em sítio anal.^11,19^ No entanto, achados do presente estudo diferem de outros estudos conduzidos em população transgênero, que identificaram uma prevalência de HPV de 19%, sendo maior entre homens transgênero (24,2%) do que em mulheres transgênero (11,8%).^20^ Quando analisados por sítio genital, foi observada maior presença de HPV em sítio anal em mulheres transgênero (48,1%) do que em homens transgênero, o que evidencia risco aumentado das mulheres em infecções anais. Além disso, a infeção de HPV em sítio cervical (37,2%) foi semelhante a estudos prévios que encontraram HPV cervical de até 25%, mas difere de outros estudos que identificaram positividade em 73% das pessoas nascidas com colo de útero.^20,21^

Não houve diagnóstico de neoplasia anal, mas encontrou-se alteração de citologias oncóticas em 23,5% das mulheres transgênero – diferentemente do que é descrito em outros estudos, que encontraram taxas de até 91% –, e tal resultado equipara mulheres transgênero a um grupo mais propício ao desenvolvimento de neoplasia anal.^22,23^ Ainda assim, a citologia anal alterada neste estudo é inferior ao encontrado em algumas populações cisgênero (25,5%), especialmente a população de homens que fazem sexo com homens (43,6%), mas similar a mulheres heterossexuais (19,4%).^13^ O mesmo ocorre com o diagnóstico de HPV anal, que se equipara a alguns cortes transversais em mulheres transgênero.^19^ Em homens transgênero, lesão pré-neoplásica cervical não influenciou nos diagnósticos de lesão pré-neoplásica anal, assim como o uso das próteses penianas também não influenciou nos diagnósticos de neoplasias intraepiteliais cervicais ou de HPV em sítio cervical. 

Além do mais, mulheres transgênero, que são tradicionalmente associadas pela literatura científica a comportamentos de riscos e alta taxa de alteração em citologias anais, não possuem protocolos ou diretrizes específicos para rastreamento de neoplasia anal.^10^ Quando perguntadas sobre a realização de exames de citologia para rastreamento de câncer genital, 78,2% das participantes negaram qualquer realização, enquanto em homens transgênero esse valor chegou a 66,7%, maior que em estudos que identificaram 42,3% de ausência nos exames de rastreamento para câncer de colo de útero.^20^

Além de alta frequência de HPV, vale ressaltar que a prevalência de infecção pela clamídia está abaixo do encontrado em estudos brasileiros, que identificaram 12% dessa infecção, e difere nos diagnósticos de gonorreia (6%), que não foi identificada nos casos avaliados neste estudo.^24^ Apesar da prevalência de tricomoníase neste estudo, não foram encontrados estudos recentes com positividade para tricomoníase na população transgênero,^8,25^ o que faz dessa informação um dado pioneiro para o estudo de IST em população transgênero.

Este estudo apresentou maioria de homens transgênero em relação a mulheres transgênero, diversamente do que já foi descrito em literatura médica.^26^ Ainda assim, os diagnósticos de IST em homens transgênero foram inferiores aos encontrados em cortes transversais anteriores,^8^ mas similares a outros que também demonstraram baixas taxas de diagnósticos entre eles,^12,26^ relacionando-se esse fato a comportamentos sexuais seguros nesse grupo, mesmo apesar da alta taxa de não uso de preservativo entre eles (55,6%). Ainda no que concerne ao impacto dos comportamentos sexuais na prevalência do HPV, observou-se que a realização de intercurso com penetração com pênis em qualquer sítio aumenta a chance de infecção pelo vírus do HPV; mas, estratificando-se por sítio de penetração, o sítio vaginal apresenta menor chance. Ademais, o uso de preservativo não protege contra a transmissão do vírus do HPV, dado bem estabelecido por estudos anteriores, que relatam a transmissão do vírus mesmo com o uso do preservativo.^27^

Pessoas transgênero são frequentemente vítimas de estigmatização e discriminação social, o que influencia muito em seu bem-estar e saúde.^28^ Mesmo que quase metade da amostra tenha relatado acesso a algum serviço de saúde previamente à aplicação do questionário de pesquisa, 46,2% relataram algum tipo de discriminação no atendimento, principalmente no distrato ao nome social e ao gênero com o qual a pessoa se identifica. Outros 44,2% declararam que não se sentiram bem orientados em relação à transição de gênero em serviços públicos de saúde. A ausência de diretrizes para acolhimento e definição de critérios para atendimento e seguimento nos níveis de atenção à saúde à população transgênero ainda é uma barreira, dificultando a atenção às pessoas transgênero em serviços de saúde e ressaltando a necessidade de quebra de barreiras, fornecimento de atendimento de qualidade e de construção de conhecimento científico acerca dessa população.^29^

A dificuldade de acesso à saúde e aos centros de pesquisa dificulta a investigação científica sobre a população transgênero, prejudicando ações de inclusão social e melhorias na qualidade de atendimento em saúde. Faz-se necessário gerar condições para a implementação de políticas efetivas e inclusivas. Abordar as barreiras enfrentadas pela população transgênero no acesso aos serviços de saúde é imperativo para promover a equidade e melhorar os resultados de saúde dessa comunidade.^2^ A implementação de estratégias inclusivas e sensíveis ao gênero é essencial para garantir que todos tenham acesso aos cuidados de saúde de qualidade, independentemente da identidade de gênero.^3^

Este estudo apresentou algumas limitações, como a própria característica do estudo transversal de medir a exposição e o desfecho ao mesmo tempo, não permitindo se estabelecer uma relação de causa e efeito; o tamanho amostral também foi relativamente pequeno, o que pode ter dificultado algumas comparações e associações. Além disso, as amostras anogenitais foram prejudicadas pela recusa dos participantes em sua coleta, seja por desconhecimento da importância da realização de exames de rastreamento, seja por se tratar de exames que poderiam desencadear sintomas disfóricos. Outra limitação em relação às amostras de sítio anal é a presença de resultados insatisfatórios, devido à hipocelularidade da região, segundo os critérios de Bethesda atualizados, o que atrapalha a análise histopatológica adequada na procura de alterações pré-neoplásicas celulares.^30^ Ademais, o estudo foi realizado durante a pandemia do vírus da covid-19, que suspendeu serviços eletivos ambulatoriais, prejudicando o número da amostra final e contribuindo para as ausências de dados em pesquisa de amostras anogenitais e IST. Entretanto, mesmo com essas limitações, este estudo representa um importante avanço em saúde da população transgênero, tendo em vista que traz dados exploratórios inéditos que podem contribuir na formulação de políticas públicas. 

Uma vez que pessoas transgênero são acompanhadas em serviço de saúde adequado e atendidas por profissionais de saúde treinados, é possível avançar em políticas de prevenção de câncer de trato anogenital e IST, aumentando assim a taxa de diagnóstico adequado e tratamento precoce. A alta prevalência de infecção genital pelo vírus HPV e de citologias anogenitais alteradas – mesmo com métodos de prevenção primário e secundário amplamente disponíveis no Sistema Único de Saúde e de conhecimento amplamente difundido na prática médica – é alarmante, e deve atrair a atenção de órgãos e políticas públicas. Apesar da descoberta de dados valiosos a respeito dessa população, espera-se que estudos mais robustos se desenvolvam, para melhor avaliação de prevalência de IST e sua associação com dados clínico-comportamentais.
